# Improved Quality of Life in Children With Cystic Fibrosis Who Received Transmembrane Conductance Regulator Modulators

**DOI:** 10.1111/apa.70498

**Published:** 2026-03-11

**Authors:** Marcus Svedberg, Frida Lundqvist, Huda Abdulahi Östrand, Christina Krantz, Per Ekman, Henrik Imberg

**Affiliations:** ^1^ Department of Paediatrics, Institute of Clinical Sciences, Sahlgrenska Academy Gothenburg University Gothenburg Sweden; ^2^ Department of Paediatrics Queen Silvia's Children's Hospital Gothenburg Sweden; ^3^ Centre for Digital Health, Sahlgrenska University Hospital Gothenburg Sweden; ^4^ Department of Paediatrics Skåne University Hospital Lund Sweden; ^5^ Department of Paediatrics Uppsala University Hospital Uppsala Sweden; ^6^ Statistiska Konsultgruppen Sweden Gothenburg Sweden; ^7^ Department of Molecular and Clinical Medicine Institute of Medicine, Sahlgrenska Academy, University of Gothenburg Gothenburg Sweden

**Keywords:** cystic fibrosis, elexacaftor tezacaftor ivacaftor, inhalation therapy, transmembrane conductance regulator modulators, treatment burden

## Abstract

**Aim:**

Children with cystic fibrosis (CF) face substantial daily treatment burdens and the effects of transmembrane conductance regulator modulators on these have not been sufficiently described. We evaluated changes in treatment burden after elexacaftor tezacaftor ivacaftor (ETI) was initiated.

**Methods:**

This prospective observational study comprised children aged six to 17 years from three Swedish paediatric CF centres. They were enrolled from 7 December 2022 to 30 June 2023, before ETI was initiated. Their health‐related quality of life, treatment burden, and adherence were assessed at baseline and at six and 12 months.

**Results:**

We studied 62 children who initiated ETI and were followed up to 12 months. The mean standard deviation (SD) treatment burden score improved by 4.9 points (95% confidence interval (CI) −0.7 to 10.6, *p* = 0.087), from 67.6 (±20.6) at baseline to 72.5 (±17.6) at 12 months. The mean daily time spent on inhalation therapy and airway clearance decreased from 91.9 (±37.3) to 70.4 (±23.6) minutes (*p* < 0.001). Mean inhalation time per session fell by 7 min (95% CI −10.5 to −3.3, *p* < 0.001) and airway clearance time by 4 min (95% CI −6.7 to −1.5, *p* = 0.003).

**Conclusion:**

Treatment burden, particularly daily inhalation and airway clearance time, decreased after ETI initiation.

## Summary

- Children with cystic fibrosis face substantial daily treatment demands, but it is unclear how transmembrane conductance regulator modulators affect treatment burden.
- We measured the treatment burden of children aged six to 17 years who received elexacaftor tezacaftor and ivacaftor, at baseline, six and 12 months.
- The mean treatment burden score improved and the time spent on inhalation therapy and airway clearance each day decreased by 7 and 4 min, respectively.

**Abbreviations**
CF: cystic fibrosis
CFQ‐R: cystic fibrosis Questionnaire—Revised
CI: confidence intervals
ETI: elexacaftor tezacaftor ivacaftor
SD: standard deviation

## 1 Introduction

Cystic fibrosis (CF) is a life‐limiting, multisystem disorder caused by mutations in the *CF transmembrane conductance regulator* gene [1, 2]. The disease leads to dehydrated and viscous secretions within the respiratory and gastrointestinal tracts, resulting in recurrent pulmonary infections, malabsorption, and progressive loss of lung function [1, 2, 3]. Advances in multidisciplinary care and pharmacological therapies have markedly improved clinical outcomes and survival. However, the daily treatment burden remains considerable for affected children and their families.

Managing CF is complex and time‐intensive. Treatment typically involves daily inhalation and airway clearance therapy, pancreatic enzyme replacement, nutritional supplementation, and regular physical activity [4]. These regimens often require one and a half to two hours per day, placing a substantial burden on children with CF and their families [5].

Parents play a crucial role in coordinating and administering treatments in paediatric CF care [6]. This responsibility adds a further layer of complexity to the overall treatment burden, as families must balance the demands of intensive medical care with the routines of everyday parenting. The burden of treatment may also vary depending on the child's age, disease severity, family structure, and the availability of healthcare support. Moreover, treatment protocols often differ across countries due to variations in medical traditions, resource availability, and healthcare infrastructure [7].

Over the past 5 years, CF treatment has advanced substantially with the introduction of highly effective CF transmembrane conductance regulator modulators such as elexacaftor tezacaftor ivacaftor (ETI). These therapies have led to significant improvements in lung function, nutritional status, and respiratory symptoms in children with CF [8, 9].

ETI was approved by the European Commission for use in individuals with CF aged 12 years and older in August 2020 and in children aged six to 11 years in January 2022. ETI was reimbursed in Sweden since 1 December 2022 for people with CF 6 years or older, with approximately 85% of children with CF being clinically eligible based on their CFTR genotype. While the clinical efficacy of ETI has been well established, its impact on the daily treatment burden remains less well understood.

The aim of this study was to assess changes in the overall treatment burden of Swedish children aged six to 17 years with CF, at baseline and six and 12 months after the initiation of ETI therapy. This included changes in treatment adherence and time spent on daily therapies.

## 2 Methods

### 2.1 Study Design and Setting

This was a prospective, observational multicentre study conducted in three paediatric CF centres in Sweden: Queen Silvia's Children's Hospital in Gothenburg, Skåne University Hospital in Lund and Uppsala University Hospital in Uppsala. Enrolment took place from 7 December 2022 to 30 June 2023, after national reimbursement approval of ETI. Each child, together with their parent, was scheduled for follow‐up at baseline, 6 and 12 months after ETI initiation.

Children aged six to 17 years with a confirmed diagnosis of CF were eligible if ETI was initiated during the enrolment period. Study participation required the involvement of both the child and one parent. Children were excluded from the analysis if ETI therapy was discontinued. No other exclusion criteria were applied.

### 2.2 Procedures

**FIGURE 1:**
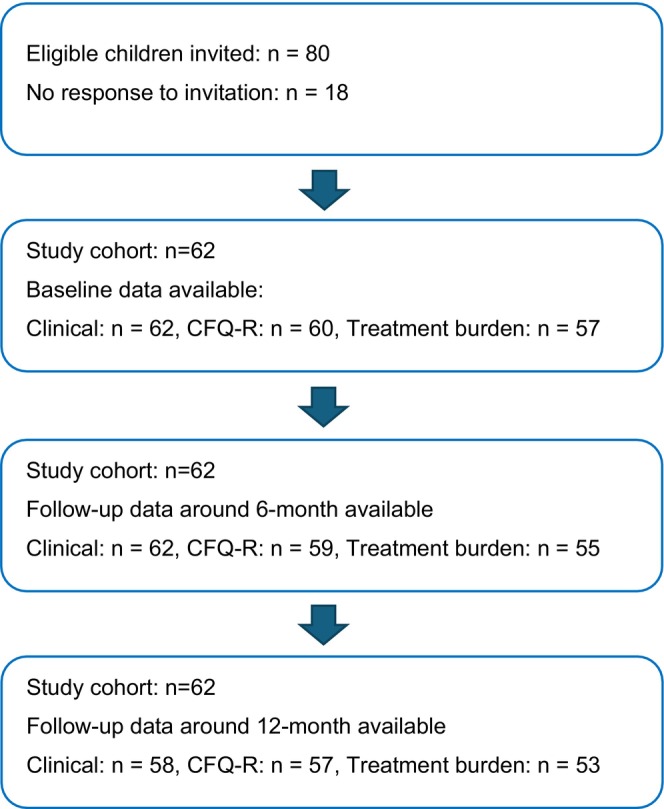
Study flow diagram showing child inclusion and the availability of clinical data, Cystic Fibrosis Questionnaire–Revised (CFQ‐R) questionnaires, and treatment burden questionnaires at baseline, around 6 months, and around 12 months after initiation of elexacaftor tezacaftor ivacaftor therapy. Clinical data on lung function and anthropometry were retrieved from the Swedish National Cystic Fibrosis Registry at the corresponding follow‐up visits.

Parents of all children, preferably together with their child, completed the treatment burden questionnaire. The CF Questionnaire–Revised (CFQ‐R) was completed by parents for children aged six to 13 years and by older children aged 14–17 years themselves, in accordance with the questionnaire's validated age‐specific versions. Both the treatment burden questionnaire and the CFQ‐R were scheduled for follow‐up at baseline, 6 and 12 months (Figure 1).

Clinical data, including lung function and anthropometric measurements, were extracted from the Swedish National CF Registry at each visit. Children were required to maintain their usual treatment regimens, including inhalation and airway clearance therapies, unless medically modified. Standard treatment included twice‐daily inhalation therapy with 4 mL hypertonic saline administered via a mesh or jet nebulizer. This was followed by airway clearance techniques, including positive expiratory pressure and huffing techniques to facilitate mucus clearance.

### 2.3 Outcome Measurements

#### 2.3.1 Health‐Related Quality of Life

Health‐related quality of life was assessed using the CFQ‐R, which was completed digitally either during a scheduled clinical visit or at home prior to the visit via a secure online link. The CFQ‐R assesses symptoms and experiences during the 14 days preceding questionnaire completion, including the baseline assessment conducted at the time when ETI initiation was planned. The CFQ‐R is a validated, disease‐specific instrument designed to capture the physical, emotional, and social impact of living with CF [10]. The analysis focused on two CFQ‐R domains, treatment burden and respiratory symptoms. The treatment burden domain evaluates the time, difficulty, and interference associated with daily CF therapies. The respiratory domain reflects symptom severity related to airway function. Domain scores range from 0 to 100, with higher scores indicating lower perceived burden or fewer symptoms. For children aged six to 13 years, the CFQ‐R was completed by one parent per child, while older children aged 14–17 years completed the questionnaire independently. Self‐report is recommended and validated for this age group in the age‐specific CFQ‐R formats. As the identical domains and scoring system were used across versions, CFQ‐R data from younger and older children were pooled for analysis.

#### 2.3.2 Treatment Burden Questionnaire

A study‐specific questionnaire was developed to explore the factors that contributed to treatment burden, because no validated Swedish tool existed to capture these aspects. It was designed by a multidisciplinary CF research team, which included physicians, physiotherapists, dietitians, and CF nurses. The questionnaire was administered digitally and completed by one parent per child, who served as the primary respondent, preferably together with their child. Each question reflected experiences during the 14 days preceding each study visit.

The questionnaire comprised 16 multiple‐choice questions. Three questions addressed the perceived time and effort associated with inhalation therapy and airway clearance techniques. One question focused on upper airway clearance, four on sleep quality, three on eating habits, and three on social participation. Adherence to inhalation and airway clearance therapies was captured by two questions. Adherence was defined as completing at least 80% of the prescribed treatments during the previous 14 days [11]. Reported treatment duration included pre‐medication, therapy administration, and cleaning procedures. Responses indicating treatment burden prompted an optional free‐text item allowing further clarification (data not presented). When support was required, questionnaire completion was assisted by CF nurses from the clinical care team.

#### 2.3.3 Clinical Measures

Clinical data were retrieved from the Swedish National CF Registry at baseline, 6 and 12 months. Lung function was assessed using forced expiratory volume in 1 s and forced vital capacity, both expressed as the percentage of predicted values calculated according to the Global Lung Function Initiative reference equations [12]. Anthropometric measurements, including weight, height, and body mass index, were converted to age‐ and sex‐standardised z‐scores based on Swedish reference data [13].

### 2.4 Statistics

Descriptive data were summarised using means and standard deviations (SDs) for continuous variables, and counts and percentages for categorical variables.

Longitudinal changes in CFQ‐R scores, treatment burden questionnaire outcomes and respiratory function from baseline to six and 12 months were analysed. Generalised least squares regression for repeated measures, with an unstructured covariance matrix, was used to account for intra‐individual correlation and missing data under the assumption of missing at random.

Results were presented as estimated marginal means and standard deviations (SD) for continuous outcomes, alongside adjusted mean differences with 95% confidence intervals (CI). For categorical outcomes, absolute percentage point changes with 95% confidence intervals are reported.

All statistical tests were two‐sided and conducted at a 5% significance level. Analyses were performed using SAS software, version 9.4 (SAS Institute Inc., North Carolina, USA).

### 2.5 Ethics

Written informed consent was obtained from the parents of children aged 6–14 years or directly from children aged 15 years or older, in accordance with Swedish ethical regulations. The study was approved by the Swedish Ethical Review Authority (number 2022–06910‐01).

## 3 Results

### 3.1 Study Population Characteristics

Between 7 December 2022 and 30 June 2023, the families of 80 children aged six to 17 years with CF were invited to take part in the study during a clinic visit when initiation of ETI was planned. Of these, 18 (22%) did not respond to the invitation to participate despite reminders, providing no further reason. The remaining 62 children met the eligibility criteria and were included in the analysis.

Demographic characteristics of the children are presented in Table 1. All 62 children remained in the study cohort throughout follow‐up. However, the availability of clinical data, CFQ‐R questionnaires, and treatment burden questionnaires varied at baseline, 6 and 12 months, as presented in Figure 1.The median follow‐up time was 12 (range 9–16) months, resulting in some variability around the nominal six‐ and 12‐month time points. Clinical data was available for 182/186 (98%) scheduled visits. During the study period, a total of 176/186 (95%) CFQ‐R surveys and 165/186 (89%) treatment burden questionnaires were completed. Of the 165 treatment burden surveys, 117 (71%) were completed collaboratively by the parent and their child.

**TABLE 1:** Demographics and clinical characteristics of the study cohort at baseline, prior to elexacaftor tezacaftor ivacaftor therapy.

	Study population (*n* = 62)
CF centre	
Gothenburg	38 (61%)
Lund	13 (21%)
Uppsala	11 (18%)
Sex male	38 (61%)
Age (years)	11.7 (3.4)
6–13 years	42 (68%)
14–17 years	20 (32%)
CF mutation	
F508del/other mutation^a^	28 (45%)
F508del/F508del	34 (55%)
Pancreatic insufficiency	60 (97%)
CF‐related diabetes	6 (10%)
CFQ‐R^b^	
Treatment burden domain (score 0–100)	65 (18)
Respiratory domain (score 0–100)	85 (14)
Lung function	
FEV_1_%	93.3 (13.9)
FVC%	97.6 (12.0)
Anthropometric z‐scores	
Weight	−0.37 (1.24)
Height	−0.45 (1.28)
Body mass index	−0.17 (1.12)
CFTR modulator therapy	
None	29 (47%)
Ivacaftor/Lumacaftor	33 (53%)
Inhalation therapy sessions	
Once daily	3 (5%)
Twice daily	59 (95%)

*Note:* Descriptive data are presented as means and standard deviations for continuous variables, and as counts and percentages for categorical variables.

Abbreviations: CF, cystic fibrosis; CFQ‐R, Cystic Fibrosis Questionnaire‐Revised; CFTR, cystic fibrosis transmembrane conductance regulator; FEV_1_%, forced expiratory volume in one second, percent predicted; FVC%, forced vital capacity, percent predicted.

^a^ Other mutation indicates any non‐F508del mutation.

^b^ CFQ‐R data were available for 60 children.

### 3.2 Changes in Treatment Burden, Respiratory Symptoms and Lung Function

Longitudinal changes in CFQ‐R scores and lung function during follow‐up are presented in Table 2. Over the 12‐month follow‐up period, the mean CFQ‐R treatment burden domain score (0–100 scale) increased from 67.6 (SD ±20.6) at baseline to 72.5 (SD ±17.6) at 12 months, corresponding to a mean improvement of 4.9 points (95% CI −0.7 to 10.6; *p* = 0.087).

**TABLE 2:** Changes in Cystic Fibrosis Questionnaire‐Revised (CFQ‐R) treatment burden and respiratory domain scores, and lung function before and 12 months after initiation of elexacaftor tezacaftor ivacaftor therapy. CFQ‐R scores range from 0 to 100, with 100 representing the best possible score.

	Baseline, mean (SD)	12 months, mean (SD)	Mean difference (95% CI)	*P*
CFQ‐R treatment burden domain	67.6 (20.6)	72.5 (17.6)	4.9 (−0.7, 10.6)	0.087
CFQ‐R respiratory domain	84.8 (8.8)	92.3 (13.9)	7.5 (3.6, 11.5)	< 0.001
FEV_1_%	93.3 (13.8)	100.5 (13.9)	7.2 (4.1, 10.2)	< 0.001
FVC%	97.6 (12.5)	101.8 (12.0)	4.2 (2.0, 6.4)	< 0.001

*Note:* Statistical analyses were performed using generalised least squares regression for repeated measures at baseline, 6 months and 12 months, accounting for intra‐individual correlation and missing data. Clinical data were available for 182 of 186 scheduled visits and 176 of 186 CFQ‐R questionnaires were completed. Descriptive data are presented as estimated marginal means and standard deviations. Mean differences are shown with 95% confidence intervals (CIs).

Abbreviations: CFQ‐R, Cystic Fibrosis Questionnaire—Revised; FEV1%, forced expiratory volume in 1 s, percent predicted; FVC%, forced vital capacity, percent predicted.

The mean CFQ‐R respiratory domain score (0–100 scale) increased from 84.8 (SD ±8.8) at baseline to 92.3 (SD ±13.9) at 12 months, corresponding to a mean improvement of 7.5 points (95% CI 3.6 to 11.5; *p* < 0.001).

### 3.3 Changes in Treatment Burden Questionnaire

Changes in treatment burden over the mean 12‐month follow‐up period are presented in Table 3. The mean (SD) estimated time spent on an inhalation therapy session fell by of 7 min (95% CI −10.5 to −3.3, *p* < 0.001), from 27 (±8) minutes to 20 (±13) minutes at follow‐up. Similarly, the mean time spent on an airway clearance therapy session fell by a mean of 4 min (95% CI −6.7 to −1.5, *p* = 0.003), decreasing from 20 (±8) minutes to 16 (±10) minutes. Mean total daily inhalation and airway clearance time (performed twice daily) decreased from 91.9 (±37.2) minutes at baseline to 70.4 (±23.6) minutes at 12 months. The model‐estimated mean reduction was −24.2 min (95% CI −33.9 to −14.4, *p* < 0.001). Adherence to both inhalation and airway clearance therapy remained high throughout the study period.

**TABLE 3:** Estimated changes in parent‐reported daily treatment burden and related behaviours in children before and after initiation of elexacaftor tezacaftor ivacaftor therapy.

	Baseline	12 months	Adjusted mean difference (95% CI)	*P*
Airway therapy				
Adherence to prescribed inhalation therapy, %	91	92	1.7 (−5.4, 8.7)	0.64
Adherence to prescribed airway clearance, %	87	87	0.1 (−10.9, 11.2)	0.98
Airway clearance replacement with physical activity, %	61	55	−6.6 (−23.7, 10.5)	0.45
Nasal rinsing, %	51	52	−1.4 (−11.5, 8.6)	0.78
Eating habits				
Mealtime conflicts, %	33	22	−10.7 (−24.6, 3.1)	0.13
Sleep patterns				
Good sleep quality, %	85	91	6.8 (−2.7, 16.4)	0.16
Nocturnal cough, %	59	34	−24.7 (−42.4, −7.0)	0.007
Daytime napping, %	36	34	−1.9 (−16.7, 12.9)	0.80
Increased daytime fatigue, %	15	12	−3.4 (−14.0, 7.2)	0.53
Social impact				
Treatment prevents activities, %	7	2	−5.1 (−12.7, 2.4)	0.18
Treatment prevents sleepovers, %	31	16	−14.5 (−27.5, −1.5)	0.029
Treatment prevents eating at a friend's house, %	19	9	−9.5 (−21.7, 2.7)	0.13

*Note:* For all categorical variables, baseline and 12‐month values represent model‐estimated percentages of respondents answering “yes” to each item. Statistical analyses were performed using generalised least squares regression for repeated measures at baseline, 6 months, and 12 months, accounting for intra‐individual correlation and missing data. Analyses included all available treatment burden questionnaire data, comprising 165 of 186 completed questionnaires across the study period. For categorical outcomes, adjusted mean differences represent absolute percentage point changes with 95% confidence intervals.

Abbreviations: CF, Cystic Fibrosis; CI, confidence interval.

Time spent eating breakfast remained stable at around 18 min (±9.4) after 12 months of ETI treatment. Time spent eating lunch and dinner showed a slight decrease from 23.9 min (±7.4) to 21.5 min (±10.0), corresponding to an estimated mean reduction of 2.4 min (95% CI −4.9 to 0.2; *p* = 0.067). The proportion of parents reporting mealtime conflicts decreased by 10 percentage points (95% CI −24.6 to 3.1, *p* = 0.13), although these changes were not statistically significant.

The questions about sleep quality remained largely unchanged over the 12‐month follow‐up period (Table 3). However, parent‐reported nocturnal cough among children decreased from 59% at baseline to 34% at 12 months, corresponding to a model‐estimated reduction of 24.7% points (95% CI −42.4 to −7.0, *p* = 0.007).

Social participation also improved, with the proportion of parents reporting reluctance to allow their child to participate in sleepovers decreasing by a model‐estimated 15 percentage points, from 30% at baseline to 16% at 12 months (95% CI −27.5 to −1.5, *p* = 0.029). There was also an improvement in parents' willingness to let their children eat at a friend's house, although this change did not reach statistical significance. No significant changes were reported in children getting sufficient sleep or experiencing daytime fatigue compared with their peers.

## 4 Discussion

This multicentre study evaluated changes in treatment burden among children six to 17 years old with CF during the first 12 months after initiation of ETI therapy. Total daily time spent on inhalation and airway clearance therapy, performed twice daily, decreased significantly by 24 min per day after 12 months. Although the treatment burden domain score improved, this change was not statistically significant. After 12 months of ETI treatment, improvements in lung function were observed, alongside fewer airway symptoms and markedly reduced nocturnal cough. Parents also reported greater willingness to allow their children to participate in social activities, such as sleepovers and eating at friends. Overall, ETI appeared to ease daily treatment demands and enhance family quality of life without alterations to prescribed treatment regimens.

Previous studies have shown that the treatment burden associated with daily CF management for children and their parents was substantial and time‐intensive [6]. While the overall CF burden, as measured by the CFQ‐R treatment burden domain, showed improvement 12 months after the introduction of ETI, the change did not reach statistical significance. CF treatment comprises several components, with inhalation and airway clearance therapy remaining the most time‐consuming aspects. This study found that the estimated duration of an inhalation therapy session decreased by an average of 7 min, from 27 to 20 min after ETI initiation. This reduction may be attributed to several factors, including improved efficiency in initiating therapy and reduced preparation time. Improved lung health may also have contributed to shorter inhalation durations, possibly due to less coughing. However, it is important to note that the actual time required for inhalation therapy may vary based on factors such as nebulizer type and condition, as well as the volume of fluid administered. Although these variables were assumed constant in this study, they were not directly measured. The duration of airway clearance therapy also decreased, possibly due to reduced or less viscous mucus production associated with overall improved lung health [14]. Consistent with previous studies, lung function and airway symptoms improved significantly following ETI introduction, with lung function normalising compared with healthy reference populations [8, 9]. After 12 months of ETI treatment, the combined daily time spent on inhalation therapy and airway clearance decreased to approximately 70 min, corresponding to an estimated reduction of almost 25 min per day.

Estimated adherence to inhalation and airway clearance therapy remained high, at approximately 90%, throughout the study period, indicating well‐established family routines. This level of adherence was relatively high compared with previous reports of lower adherence in CF treatment regimens [11]. Despite these improvements, inhalation and airway clearance therapy continued to represent a major component of daily treatment burden, which may explain why the overall treatment burden score did not change significantly. Identifying effective ways to further simplify the treatment burden for people with CF remained a top priority, even after the introduction of ETI [15]. Ongoing clinical trials, such as the Cystic Fibrosis—Streamlining Treatments Or Reducing Medications trial, have addressed this question. This randomised, open‐label trial assessed changes in respiratory function for children with CF treated with ETI following rationalisation of nebulised mucoactive therapies. The trial investigated whether inhalation therapy could be reduced or eliminated without compromising CF lung health. These efforts reflect the ongoing pursuit of balancing treatment efficacy with quality of life for children with CF and their families.

Although nocturnal cough decreased markedly, from approximately 60% of children at baseline to 35% after 12 months, this potential indicator of improved sleep was not reflected in the other reported sleep outcomes. Overall sleep quality and daytime alertness remained high throughout the study period, which may partly reflect the subjective nature of these assessments. Previous studies have reported higher rates of sleep disturbance in children with CF, possibly related to poorer lung health, as sleep quality is closely linked to pulmonary function [16, 17]. The observed reduction in nocturnal cough may suggest that children receiving ETI were less frequently affected by airway infections. However, seasonal variations in viral illness could also have contributed. Further research is needed to confirm these findings.

Beyond measurable reductions in treatment time, families also described broader long‐term adaptations to daily care routines. ETI was associated not only with shorter and less burdensome basic treatments but also with greater flexibility in everyday life. Parents reported an increased willingness to allow sleepovers and meals at friends' homes, which likely reflects both improved child well‐being and a reduced need for tightly structured treatment schedules. Together, these findings suggest that the benefits of ETI extended beyond medical outcomes to positively influence family routines, autonomy, and the social and emotional dimensions of life for children six to 17 years with CF.

## 5 Strengths and Limitations

A major strength of this study was its multicentre, prospective design, capturing real‐world experiences from a large proportion of Swedish children initiating ETI. The inclusion of parent perspectives alongside clinical data provided a more comprehensive understanding of how treatment burden evolved in everyday life. The use of repeated measures over 12 months also helped to mitigate the impact of missing data, ensuring a more complete representation of longitudinal changes over time.

However, several limitations should be acknowledged. The sample size was relatively small, which may have limited statistical power and the generalisability of the findings. The study‐specific treatment burden questionnaire, although developed collaboratively by experienced CF clinicians, was not formally validated and relied primarily on parent‐reported data, thereby introducing potential recall or reporting bias. The absence of a comparator group not receiving ETI precludes conclusions about causality and the 12‐month follow‐up period may not have captured longer‐term adaptations in treatment routines and family life. Finally, although the multicentre design enhanced external validity, the findings may not be generalisable to settings with different treatment practices or healthcare systems.

## 6 Conclusion

Twelve months of ETI therapy in children 6–17 years was associated with reduced daily treatment time, improved lung function, and fewer airway symptoms. These findings suggest that ETI may ease treatment burden and support improvements in everyday life for children with CF and their families.

## Author Contributions

Marcus Svedberg: Conceptualization (lead); Funding acquisition (lead); Project administration (lead); Investigation (lead); Methodology (lead); Writing – original draft (lead); Writing – review and editing (equal). Huda Abdulahi Östrand: Conceptualization (supporting); Investigation (supporting); Writing – review and editing (equal). Christina Kranz: Conceptualization (supporting); Investigation (supporting); Writing – review and editing (equal). Frida Lundquist: Investigation (supporting); Writing – review and editing (equal). Per Ekman: Formal analysis (lead); Writing – review and editing (equal). Henrik Imberg: Conceptualization (supporting); Formal analysis (supporting); Writing – review and editing (equal).

## Funding

This work was supported by Drottning Silvias barnsjukhus.

## Disclosure

A large language model was used solely for linguistic support, including grammar and spelling checks. No generative AI tools were used for content creation, data analysis, interpretation, or any other part of the scientific work.

## Conflicts of Interest

The authors declare no conflicts of interest.

## Data Availability

The data that support the findings of this study are available on request from the corresponding author. The data are not publicly available due to privacy or ethical restrictions.
